# Rice LSD1-like Genes: Genome-Wide Characterization and Evidence Linking OsLSD3 to Plant Height

**DOI:** 10.3390/plants15172554

**Published:** 2026-08-22

**Authors:** Xiaojia Chen, Yuanyuan Tan

**Affiliations:** 1 State Key Laboratory of Rice Biology and Breeding, Zhejiang Provincial Key Laboratory of Crop Germplasm Innovation and Exploitation, The Advanced Seed Institute, Zhejiang University, Hangzhou 310058, China; 22416207@zju.edu.cn; 2 Xishan Modern Agriculture Joint Research Center, Zhejiang University-Wuxi, Wuxi 214117, China

**Keywords:** agronomic traits, bioinformatics analysis, gene family, LSD1-like, *Oryza sativa*, subcellular localization

## Abstract

LSD1-like zinc-finger proteins participate in programmed cell death, redox homeostasis, and stress responses in plants, but their functional diversification and contributions to agronomic variation in rice remain poorly defined. This study aimed to characterize the rice LSD1-like gene family and evaluate the potential agronomic roles of selected members, with particular emphasis on *OsLSD3*. Genome-wide analyses were integrated with *OsLSD3* natural variation and haplotype analyses in 4666 rice accessions, CRISPR/Cas9 mutant phenotyping in the ZH11 background, and subcellular localization assays. Seven LSD1-like genes were identified and showed substantial divergence in protein architecture, gene organization, promoter cis-element profiles, and tissue- and stress-responsive expression. *OsLSD3* formed six population-structured haplotypes, and two common Japonica haplotypes differed significantly in plant height. Consistently, two independent *oslsd3* mutant lines were taller than the wild type, whereas additional changes in grain-related traits were line-specific. *OsLSD2* and *OsLSD3* localized mainly to the nucleus, while *OsLSD4* was predominantly nuclear with weak cytoplasmic localization. These results identify *OsLSD3* as the strongest candidate among the examined members for further investigation of plant height- and grain-related traits, while *OsLSD2* and *OsLSD4* represent additional candidates for grain-trait regulation. Further validation using additional alleles and environments is required.

## 1. Introduction

Plants are continuously exposed to diverse biotic and abiotic stresses and rely on coordinated immune and stress-response networks for adaptation [1,2]. Programmed cell death (PCD) is a genetically controlled process involved in both plant development, such as xylem and aerenchyma formation, and defense against pathogens, particularly hypersensitive response (HR)-mediated localized cell death [3]. Following the recognition of pathogen effectors or related molecular patterns, plants rapidly activate HR, a localized form of PCD that limits nutrient acquisition by biotrophic pathogens and restricts their spread through the rapid death of infected cells [4]. These responses are typically accompanied by rapid accumulation of reactive oxygen species (ROS), including hydrogen peroxide and superoxide anions, as well as nitric oxide [5], with ROS both directly inhibiting pathogen growth and acting as redox signals to activate salicylic acid- and jasmonic acid-related defense pathways and induce pathogenesis-related genes [6]. Excessive ROS accumulation can cause oxidative stress, damaging lipids, proteins, and DNA and ultimately leading to irreversible cellular injury [7]. Therefore, plants must maintain intracellular ROS homeostasis through precise redox regulation and ROS-scavenging systems to prevent the excessive amplification of PCD signals [8].

In Arabidopsis, LESION SIMULATING DISEASE 1 (LSD1), a negative regulator of plant cell death, contains three conserved C2C2-type zinc-finger domains and modulates superoxide anion (O_2_•^−^)-associated signaling to restrict excessive cell death propagation [9,10]. Unlike classical DNA-binding transcription factors, LSD1 acts as a condition-dependent co-regulator and protein-interaction scaffold integrating ROS, light, chloroplast, and immune signals to restrict cell death [10,11]. In lsd1 loss-of-function mutants, plants are hypersensitive to cell death induced by various biotic and abiotic stimuli, including high light, long-day conditions, pathogen-related elicitation, and ultraviolet radiation. These mutants fail to restrict the expansion of necrotic lesions and eventually develop a typical runaway cell death (RCD) phenotype [9,10,12]. LSD1 can interact with AtbZIP10 and retain it outside the nucleus, thereby preventing the activation of death-associated transcriptional programs [13]. LSD1 also interacts with catalases, including CAT3, and participates in the regulation of H_2_O_2_ homeostasis and hypersensitive response-related cell death [14]. In addition, cell death in lsd1 mutants under photooxidative stress is closely associated with chloroplast-derived ROS signaling and the redox status of the plastoquinone pool, suggesting that LSD1 may participate in crosstalk between light acclimation and immune responses [10,15]. Genetic studies further demonstrated that ENHANCED DISEASE SUSCEPTIBILITY 1 (EDS1) and PHYTOALEXIN DEFICIENT 4 (PAD4) are essential immune regulators required for RCD development in lsd1 mutants [12,16]. During red light-induced PCD, LSD1 and ELONGATED HYPOCOTYL 5 (HY5) antagonistically regulate EDS1-dependent ROS and salicylic acid (SA) signaling, thereby jointly modulating the progression of cell death [17]. Overall, LSD1 maintains the boundary of plant cell death under photooxidative stress, pathogen infection, and other environmental pressures through protein–protein interactions, redox homeostasis regulation, and the integration of immune signaling networks.

Previous studies have provided important insights into the rice LSD1-like family. An early comparative study examined the phylogenetic history, domain evolution, and functional divergence of plant LSD1-like proteins using rice genome sequences [18]. Subsequent gene-specific studies showed that OsLSD1 participates in programmed cell death and callus differentiation [19], OsLOL1 (LSD ONE LIKE 1) promotes seed germination through gibberellin biosynthesis [20], OsLOL2 is associated with growth and disease resistance, and OsLOL5 contributes to saline–alkaline and oxidative stress tolerance [21,22]. LSD1-like proteins have also been implicated in interactions with rice metacaspases and pathogen effectors [23]. Nevertheless, previous studies have largely focused either on evolutionary analysis or on the functions of individual genes, and an integrated assessment connecting updated family-wide characteristics with natural variation and comparative loss-of-function phenotypes remains limited. The working hypothesis was that evolutionary and regulatory divergence within the rice LSD1-like family is accompanied by functional specialization that can be detected through distinct expression, natural variation, and mutant phenotype patterns. To test this hypothesis, the rice LSD1-like family was re-evaluated using phylogenetic, structural, collinearity, promoter, and expression analyses, and these analyses were integrated with experimental subcellular localization and CRISPR/Cas9 mutant phenotyping. By connecting family-wide divergence with population-level variation and loss-of-function phenotypes, this study provides a framework for prioritizing LSD1-like genes for further mechanistic investigation of plant architecture and grain-related traits.

## 2. Results

### 2.1. Genome-Wide Identification and Characterization of LSD1-like Gene Family Members in Rice

Based on the whole-genome protein sequences of the japonica rice cultivar Nipponbare, seven LSD1-like family members were identified in this study, namely OsLSD1–OsLSD4, OsLOL1, OsLOL2, and OsLOL5. Analysis of their physicochemical properties revealed considerable variation among these proteins. The predicted molecular weights ranged from 6.85 kDa for OsLSD4 to 151.72 kDa for OsLOL5. The theoretical isoelectric points ranged from 3.98 to 9.21, with OsLSD4 and OsLOL5 predicted to be acidic proteins, whereas the remaining members were generally basic. The instability index indicated that OsLSD3 and OsLOL1 were predicted to be stable proteins, while the other five members were classified as unstable. The grand average of hydropathicity (GRAVY) values ranged from −0.302 to 0.557, suggesting that OsLSD3 and OsLOL5 were hydrophilic, whereas the remaining members showed weak to moderate hydrophobicity. Subcellular localization prediction showed differences between the two prediction programs. Plant-mPLoc predicted most members to localize to the cell membrane, except OsLSD4 and OsLOL5, which were predicted to localize to the chloroplast and nucleus, respectively. In contrast, WoLF PSORT predicted most members to localize to the chloroplast, while OsLSD4 was predicted to localize to the mitochondrial inner membrane and OsLOL5 to the nucleus. Notably, OsLOL5 was consistently predicted to be nuclear-localized by both programs. Signal peptide prediction showed that none of the seven proteins contained a typical signal peptide, suggesting that these proteins may not enter the classical secretory pathway (Table S1).

### 2.2. Phylogenetic Classification of LSD1-like Proteins from Representative Plant Species

**Figure 1:**
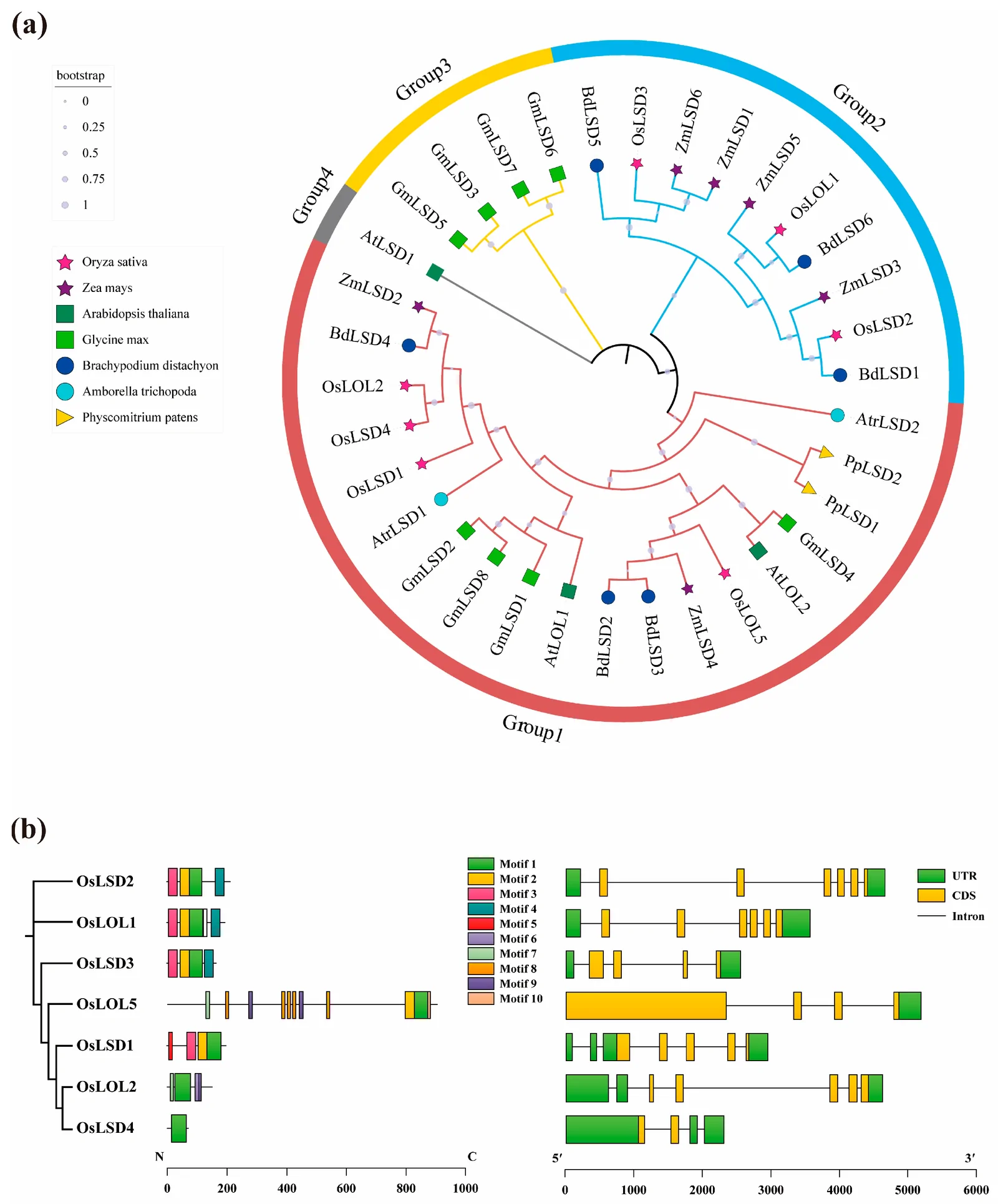
Phylogenetic relationships, conserved motif composition, and gene structures of LSD1-like family members in rice and representative plant species. (**a**) Phylogenetic analysis of LSD1-like proteins from *Oryza sativa*, *Zea mays*, *Arabidopsis thaliana*, *Glycine max*, *Brachypodium distachyon*, *Amborella trichopoda*, and *Physcomitrium patens*. The phylogenetic tree was constructed based on the full-length amino acid sequences of LSD1-like proteins. Different symbols indicate proteins from different species. Branch colors and the outer colored ring delineate the four phylogenetic groups, and node sizes represent bootstrap support values. (**b**) Phylogenetic relationships, conserved motif composition, and exon–intron structures of the seven rice LSD1-like family members. The left panel shows the phylogenetic relationships among the encoded proteins. The middle panel shows the distribution of ten conserved motifs, with each motif represented by a different colored box. The right panel shows the gene structures, in which green boxes represent untranslated regions (UTRs), yellow boxes represent coding sequences (CDSs), and gray lines represent introns. The scales indicate protein length in amino acids and genomic length in base pairs, respectively.

To examine the evolutionary relationships of LSD1-like proteins, a phylogenetic tree was constructed using homologous protein sequences from *rice*, *maize*, *Arabidopsis thaliana*, *soybean*, *Brachypodium distachyon*, *Amborella trichopoda*, and *Physcomitrium patens*. The proteins were classified into four phylogenetic groups (Figure 1a).

Group 1 was the largest group and contained four rice members, OsLSD1, OsLSD4, OsLOL2, and OsLOL5. This group also included proteins from all seven examined species, including the bryophyte P. patens and the early-diverging angiosperm A. trichopoda. Group 2 contained the remaining three rice members, OsLSD2, OsLSD3, and OsLOL1, together with homologs from maize and B. distachyon. No dicotyledonous, Amborella, or bryophyte proteins were assigned to this group. Group 3 consisted exclusively of soybean proteins, whereas Group 4 contained only AtLSD1 from A. thaliana.

The distribution of the rice proteins between Groups 1 and 2 indicates that the rice LSD1-like family comprises at least two evolutionarily distinct lineages. The broad taxonomic representation of Group 1 is consistent with evolutionary conservation, whereas the restriction of Group 2 to grass species indicates divergence within Poaceae. The soybean-specific Group 3 and the isolated position of AtLSD1 in Group 4 further illustrate lineage-associated diversification of LSD1-like proteins.

### 2.3. Conserved Motif Composition and Gene Structure of Rice LSD1-like Members

An integrated analysis of the phylogenetic relationships, conserved motifs, and gene structures of the seven rice LSD1-like members revealed both subgroup-level conservation and substantial structural variation (Figure 1b). In the rice-specific phylogenetic tree, OsLSD2, OsLOL1, and OsLSD3 clustered together, whereas OsLOL2 and OsLSD4 formed a closely related pair, with OsLSD1 positioned on an adjacent branch. OsLOL5 was separated from these compact subclusters.

Ten conserved motifs were identified in the seven proteins. OsLSD2, OsLOL1, and OsLSD3 displayed similar motif compositions and shared the conserved arrangement of Motifs 3, 2, 1, and 4. OsLSD1 contained Motifs 5, 3, 2, and 1, whereas OsLOL2 and OsLSD4 contained fewer motifs. In particular, only Motif 1 was detected in OsLSD4. By contrast, OsLOL5 was the longest family member and displayed a more complex motif composition, with several additional motifs absent from most other proteins.

The exon–intron organizations also differed among the seven genes. OsLSD2 and OsLOL1 exhibited relatively complex multi-exon structures, although their exon lengths and intron arrangements were not identical. OsLSD3 had a comparatively shorter genomic structure than OsLSD2 and OsLOL1. OsLOL5 occupied the largest genomic interval and contained a markedly extended coding region, consistent with its greater protein length and more complex motif composition. OsLSD1, OsLOL2, and OsLSD4 also differed in exon number, untranslated-region organization, and intron length despite their relatively close phylogenetic positions. These results demonstrate that closely related rice LSD1-like members retain partially conserved motif patterns, while variation in protein length, motif composition, and gene architecture distinguishes individual family members.

### 2.4. Chromosomal Distribution of LSD1-like Gene Family Members in Rice

**Figure 2:**
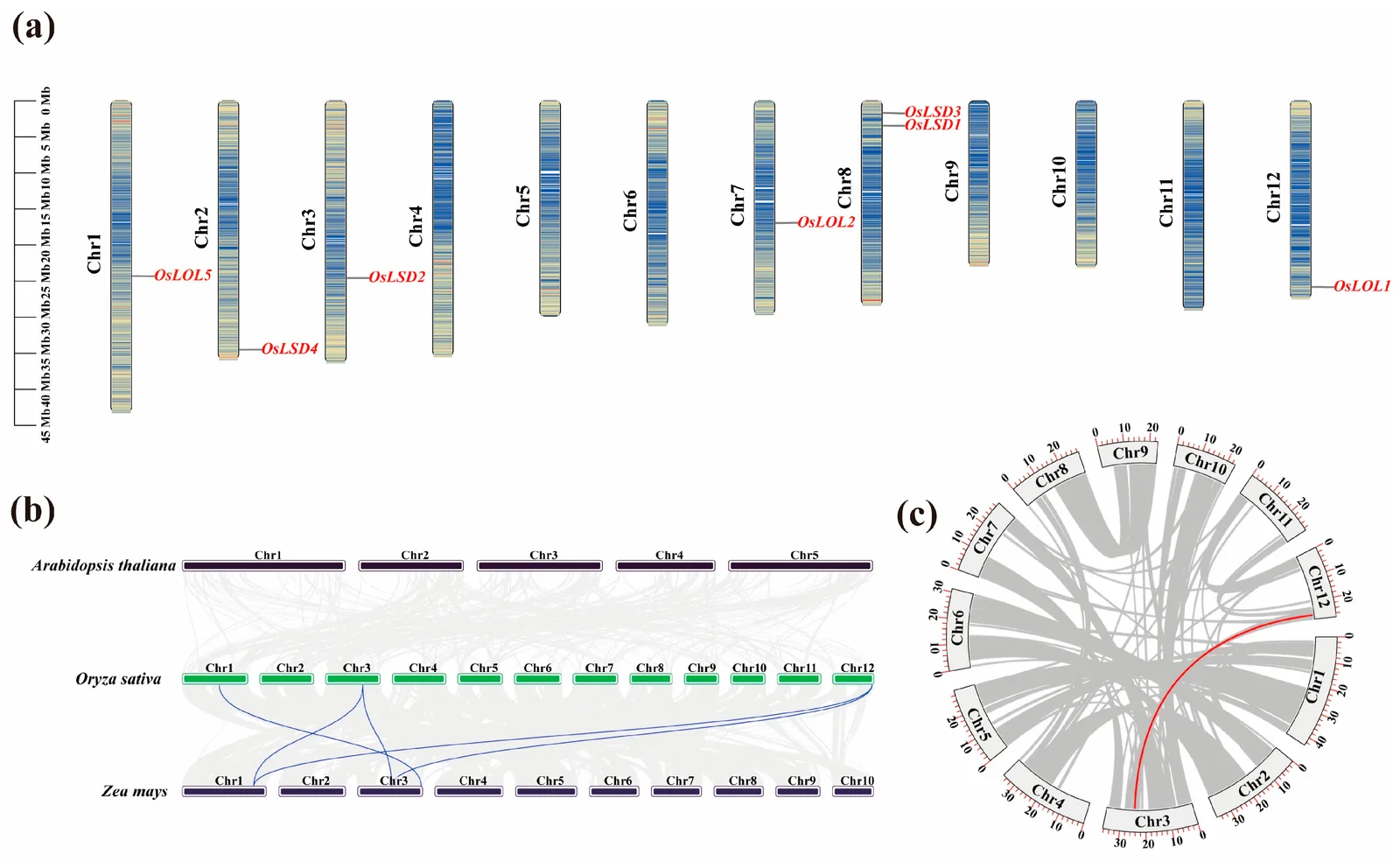
Chromosomal distribution and synteny analysis of LSD1-like genes in rice. (**a**) Chromosomal distribution of the seven LSD1-like genes in the rice genome. Chromosome numbers are indicated beside each chromosome, and the scale on the left represents chromosome length in megabases (Mb). The color gradient along each chromosome represents gene density, and the positions of LSD1-like genes are labeled in red. (**b**) Interspecific synteny analysis among *Arabidopsis thaliana*, *Oryza sativa*, and *Zea mays*. Rice was used as the reference species. Gray lines represent genome-wide syntenic blocks, whereas blue lines highlight interspecific syntenic relationships involving rice LSD1-like genes. (**c**) Intraspecific synteny analysis of rice LSD1-like genes. The 12 rice chromosomes are arranged in a circular layout. Gray ribbons represent genome-wide syntenic blocks, and the red line highlights the syntenic gene pair OsLSD2–OsLOL1. The chromosome scales are shown in megabases (Mb).

The seven rice LSD1-like genes were unevenly distributed on six chromosomes (Figure 2a). OsLOL5, OsLSD4, OsLSD2, OsLOL2, and OsLOL1 were located on chromosomes 1, 2, 3, 7, and 12, respectively, whereas OsLSD1 and OsLSD3 were both located near the upper region of chromosome 8. No LSD1-like genes were identified on the remaining six chromosomes.

### 2.5. Intraspecific and Interspecific Collinearity Analysis of the Rice LSD1-like Gene Family

Interspecific collinearity analysis identified several conserved LSD1-like gene pairs between rice and maize, whereas no corresponding collinear pairs were detected between rice and Arabidopsis thaliana (Figure 2b). Within the rice genome, only one collinear pair, OsLSD2–OsLOL1, was identified between chromosomes 3 and 12 (Figure 2c). These results indicate limited gene duplication within the rice LSD1-like family and greater syntenic conservation between rice and maize.

### 2.6. Prediction of Promoter Cis-Acting Elements in the Rice LSD1-like Gene Family

**Figure 3:**
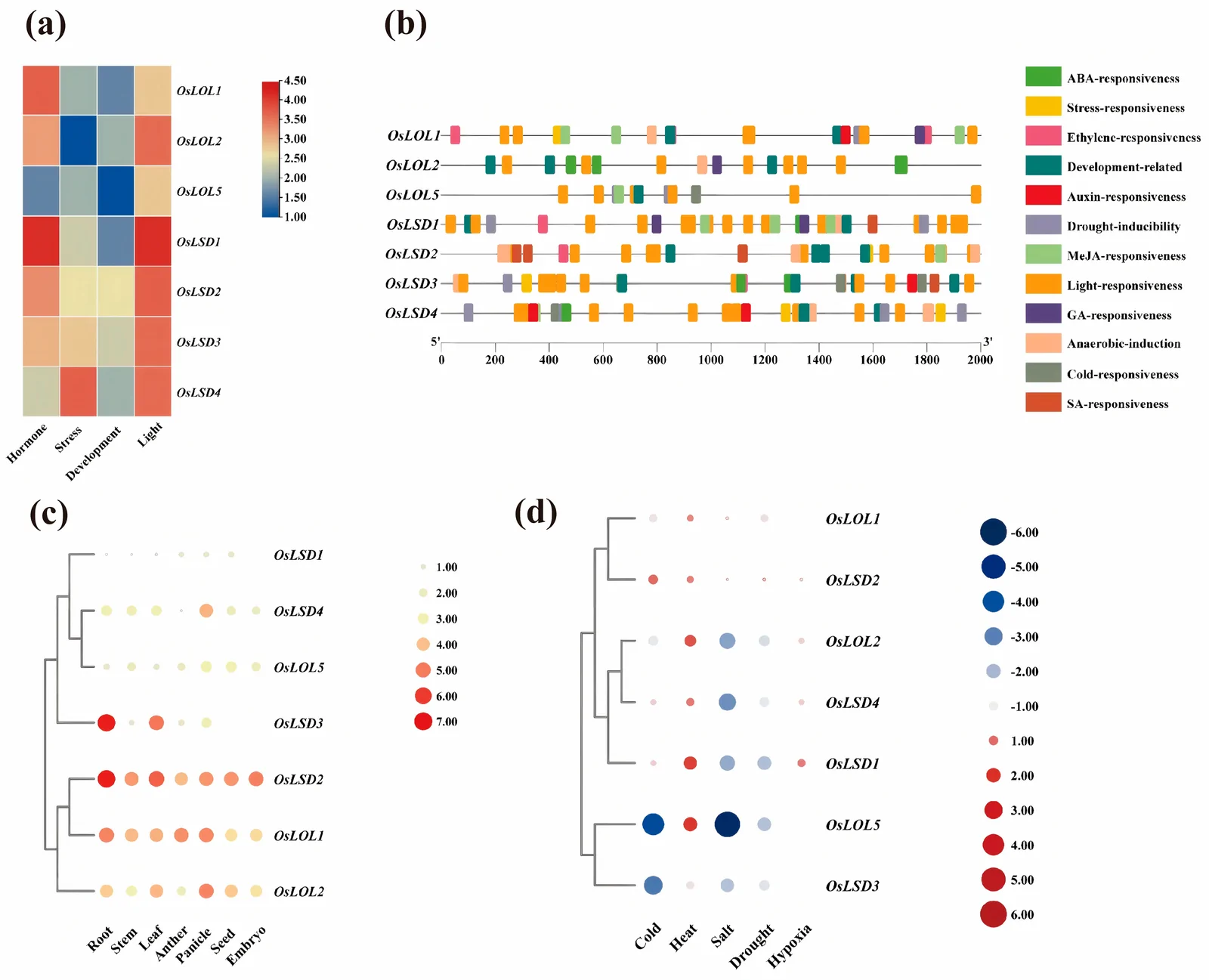
Cis-acting element distribution and expression profiles of rice LSD1-like genes. (**a**) Heatmap showing the relative abundance of cis-acting elements in the promoter regions of the seven rice LSD1-like genes. The predicted cis-acting elements were grouped into four functional categories, including hormone responsiveness, stress responsiveness, development-related elements, and light responsiveness. The color scale indicates the relative abundance of each category in individual genes. (**b**) Distribution of cis-acting elements in the 2000-bp promoter regions upstream of the start codon of the seven rice LSD1-like genes. Different colored boxes indicate different types of cis-acting elements. The scale indicates the position within the promoter region in base pairs. (**c**) Expression patterns of the seven rice LSD1-like genes in different tissues, including root, stem, leaf, anther, panicle, seed, and embryo. Circle color and size represent the relative expression level, with larger circles and deeper colors indicating higher expression. Hierarchical clustering on the left shows similarities in tissue expression patterns among genes. (**d**) Expression changes in rice LSD1-like genes under different abiotic stress treatments, including cold, heat, salt, drought, and hypoxia. Circle color indicates the direction of expression change, with red indicating upregulation and blue indicating downregulation, and circle size represents the absolute value of the normalized expression change. Hierarchical clustering on the left shows similarities in stress-responsive expression patterns among genes.

Putative cis-acting elements predicted within the 2-kb upstream regions of the seven rice LSD1-like genes were grouped into hormone-, stress-, development-, and light-responsive categories (Figure 3a). Predicted light-responsive elements were the most widely distributed, whereas the predicted abundance of hormone- and stress-responsive elements varied among genes. OsLSD1 was predicted to contain relatively more hormone-responsive elements, whereas OsLSD4 showed a higher predicted abundance of stress-responsive elements. The analysis also identified putative elements associated with responses to abscisic acid, methyl jasmonate, ethylene, auxin, gibberellin, salicylic acid, drought, cold, and anaerobic conditions (Figure 3b), suggesting potential differences in promoter regulatory architecture among family members.

### 2.7. Expression Pattern Analysis of Rice LSD1-like Genes in Different Tissues and Under Abiotic Stress Conditions

The seven LSD1-like genes exhibited distinct expression patterns across roots, stems, leaves, anthers, panicles, seeds, and embryos (Figure 3c). OsLSD2 and OsLOL1 were broadly expressed in multiple tissues, whereas OsLSD3 showed its highest expression in roots. OsLSD1 was expressed at relatively low levels, while the remaining genes displayed moderate or tissue-dependent expression.

Under cold, heat, salt, drought, and hypoxia treatments, the family members showed different transcriptional responses (Figure 3d). OsLOL5 exhibited the strongest decreases under cold and salt stress, and OsLSD3 was also downregulated under cold stress. OsLSD1, OsLOL2, and OsLSD4 were induced by heat but repressed by salt treatment, whereas responses to drought and hypoxia were generally weaker.

### 2.8. Natural Variation and Haplotype Structure of OsLSD3 and Their Association with Plant Height

**Figure 4:**
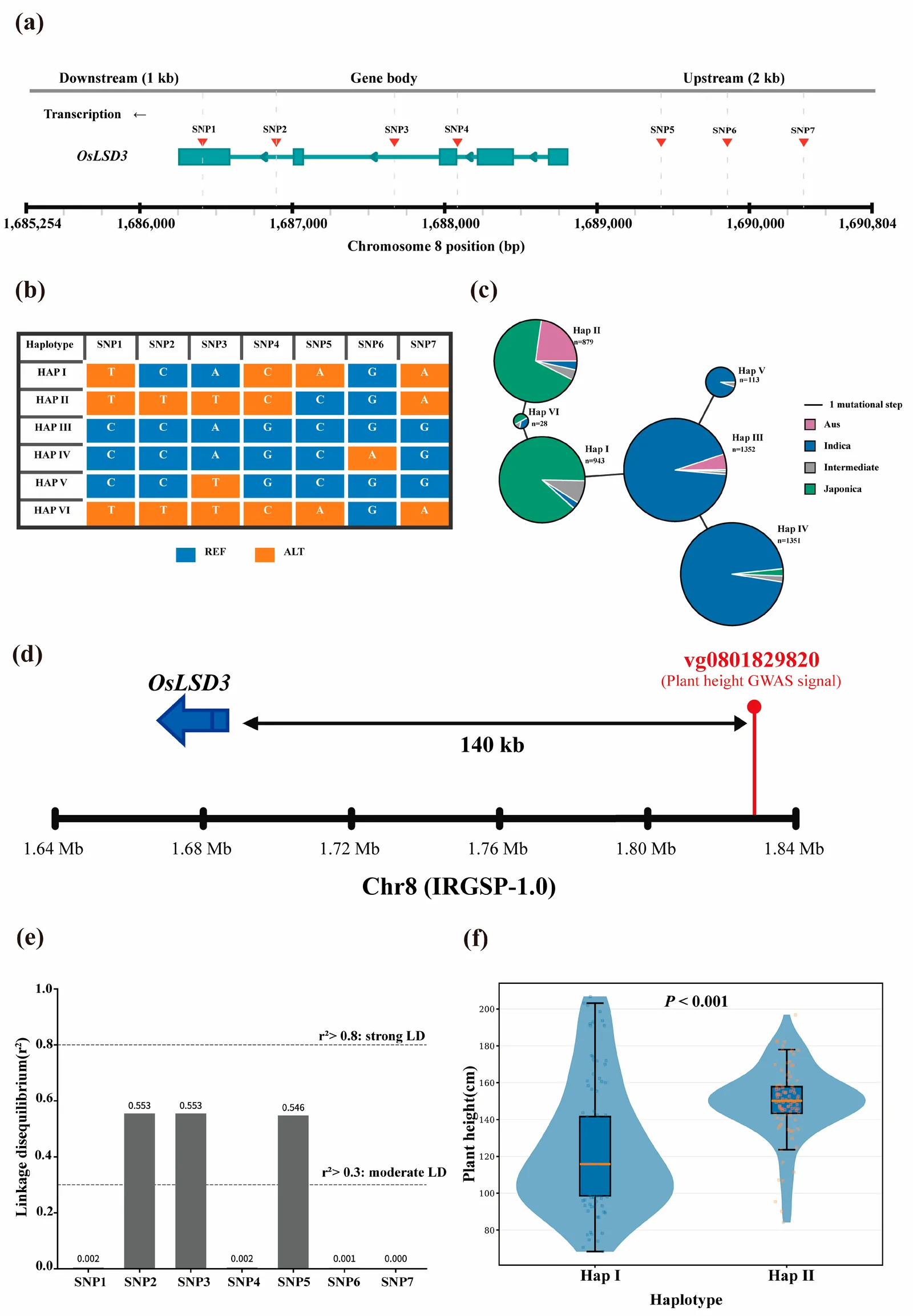
Natural variation, haplotype structure, and association of OsLSD3 haplotypes with plant height in rice. (**a**) Gene structure of OsLSD3 and positions of the seven representative core SNPs. Filled boxes indicate exons, connecting lines indicate introns, and arrows indicate the reverse-strand transcriptional orientation. Red triangles indicate SNP1–SNP7. (**b**) Allelic matrix of the six OsLSD3 haplotypes, Hap I–Hap VI, identified using the Haplotype Network Analysis module of RiceVarMap v3.0. Blue and orange cells indicate the Nipponbare reference (REF) and alternative (ALT) alleles, respectively. Letters within the cells indicate the nucleotide at each SNP, and haplotype sequences are read from SNP1 to SNP7. SNP1–SNP7 correspond to vg0801686408, vg0801686892, vg0801687667, vg0801688081, vg0801689418, vg0801689852, and vg0801690354, respectively. (**c**) OsLSD3 haplotype network and population composition generated using the Haplotype Network Analysis module of RiceVarMap v3.0 based on 4666 rice accessions. Each node represents a haplotype identified from the seven representative core SNPs. Node area is proportional to the number of accessions assigned to each haplotype, and pie-chart sectors indicate the proportions of Indica, Japonica, Aus, and Intermediate accessions. Branch length represents mutational distance across the seven SNPs, with the scale bar corresponding to one SNP difference. The network represents haplotype similarity and does not imply ancestor–descendant relationships. (**d**) Physical relationship between OsLSD3 and the plant-height GWAS lead SNP vg0801829820 on chromosome 8. The lead SNP is located approximately 140 kb transcriptionally upstream of OsLSD3 and was significantly associated with plant height in the RiceVarMap Japonica panel (*p* = 3.06 × 10^−22^). (**e**) Pairwise linkage disequilibrium between vg0801829820 and the seven OsLSD3 haplotype-defining SNPs in 1497 Japonica accessions. Linkage disequilibrium was quantified using r^2^ calculated from the observed eight-locus haplotype frequencies. (**f**) Plant-height distributions of Japonica accessions carrying OsLSD3 Hap I and Hap II. Violin plots show the overall phenotype distributions, embedded box plots indicate the median and interquartile range, and jittered dots represent individual accessions. Hap I and Hap II included 109 and 95 accessions, respectively. The difference between the two haplotypes was evaluated using Welch’s two-sample t-test and further validated using the Mann–Whitney U test. *p* < 0.001.

A total of 110 natural variants were retrieved from the OsLSD3 gene body and its flanking regions. After filtering, seven representative core single-nucleotide polymorphisms (SNPs) were selected, including one SNP in the 3′ untranslated region (3′ UTR), two intronic SNPs, one splice-region/intronic SNP, and three transcriptional upstream SNPs. These variants provided representative coverage of the OsLSD3 gene body and its upstream regulatory region (Figure 4a).

The allelic combinations of the seven core SNPs defined six haplotypes, Hap I–Hap VI, among 4666 rice accessions (Figure 4b). Hap III and Hap IV were the most frequent, accounting for 28.98% and 28.95% of the accessions, respectively, whereas Hap I and Hap II accounted for 20.21% and 18.84%, respectively. Together, these four major haplotypes represented 96.98% of all accessions. Hap V and Hap VI occurred at substantially lower frequencies of 2.42% and 0.60%, respectively (Table 1).

The haplotype network showed that Hap III and Hap IV differed only at SNP6, Hap III and Hap V differed only at SNP3, and Hap II and Hap VI differed only at SNP5, indicating that several low-frequency haplotypes were genetically close to common haplotype backgrounds (Figure 4c). Population-composition analysis showed that Hap I and Hap II were predominantly associated with Japonica, which accounted for 88.65% and 69.74% of these haplotypes, respectively. Hap II also contained 22.75% Aus accessions. In contrast, Hap III, Hap IV, and Hap V were strongly enriched in Indica, with proportions of 93.64%, 95.78%, and 94.69%, respectively, whereas Hap VI showed a mixed population composition (Figure 4c; Table 1). The distributions of the four major haplotypes differed significantly among rice population groups (χ^2^ = 4322.86, df = 9, *p* < 0.001; Cramér’s V = 0.564), indicating a strong association between OsLSD3 haplotypes and rice population structure.

In the RiceVarMap Japonica genome-wide association study (GWAS) panel, the plant-height lead SNP vg0801829820 was located approximately 140 kb transcriptionally upstream of OsLSD3 and was significantly associated with plant height (*p* = 3.06 × 10^−22^) (Figure 4d). In 1497 Japonica accessions, the lead SNP showed moderate linkage disequilibrium with SNP2, SNP3, and SNP5, with r^2^ values of 0.553, 0.553, and 0.546, respectively. In contrast, its r^2^ values with SNP1, SNP4, SNP6, and SNP7 were close to zero (Figure 4e). These results indicate that the plant-height-associated SNP was linked to only part of the OsLSD3 haplotype-defining SNP panel.

Plant-height comparison showed that the 109 Hap I accessions had a mean plant height of 123.63 cm and a median of 115.80 cm, whereas the 95 Hap II accessions had a mean plant height of 149.68 cm and a median of 150.20 cm (Figure 4f; Table 2). The mean plant height of Hap II was 26.05 cm greater than that of Hap I. Both Welch’s two-sample t-test and the Mann–Whitney U test indicated a highly significant difference between the two haplotypes (*p* < 0.001).

### 2.9. Agronomic Trait Analysis of Rice LSD1-like Gene Mutants

Sequencing confirmed seven homozygous mutant lines for four LSD1-like genes (Table 3). The *oslol1-1* and *oslol1-2* lines carried a 1-bp deletion and insertion, respectively, whereas *oslol1-3* contained a 16-bp deletion. The *oslsd2-1*, *oslsd3-1*, and *oslsd3-2* lines each carried a 1-bp insertion, while *oslsd4-1* contained a 16-bp deletion.

**Figure 5:**
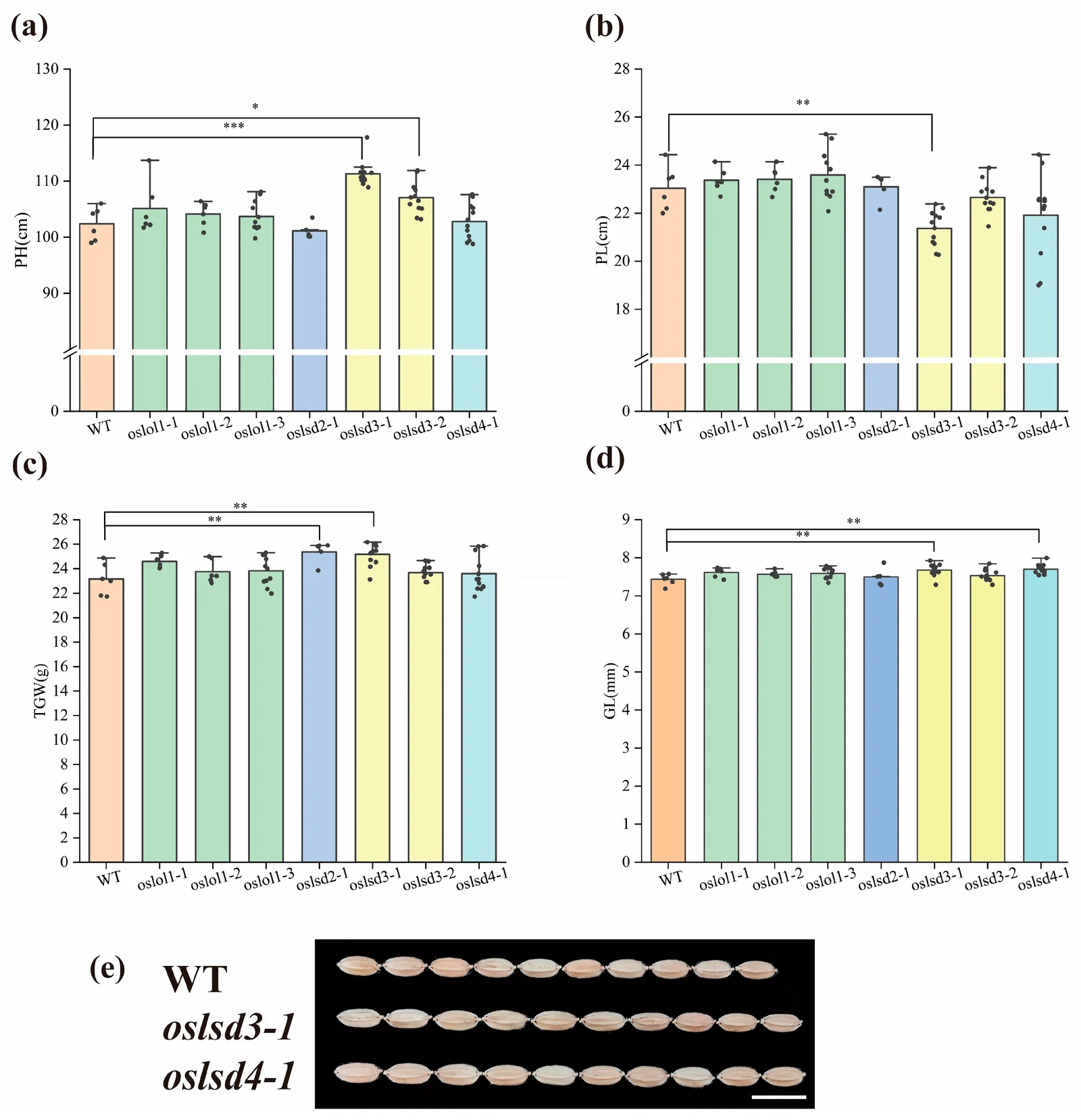
Agronomic trait analysis of wild-type Zhonghua 11 and rice LSD1-like mutant lines. (**a**–**d**) Comparison of major agronomic traits between wild-type Zhonghua 11 (ZH11/WT) and the indicated LSD1-like mutant lines, including plant height (PH, (**a**)), panicle length (PL, (**b**)), thousand-grain weight (TGW, (**c**)), and grain length (GL, (**d**)). Bars represent the mean ± SE, and dots indicate individual biological samples. Statistical significance was determined by one-way ANOVA followed by Dunnett’s test, using WT as the control. Asterisks indicate significant differences compared with WT: * *p* < 0.05, ** *p* < 0.01, and *** *p* < 0.001. (**e**) Representative grain morphology of WT, *oslsd3-1*, and *oslsd4-1*. Scale bar = 1 cm.

Agronomic trait analysis identified significant differences between several mutant lines and the wild type (WT) (Table 4 and Tables S9 and S10). Plant height increased from 102.38 ± 1.20 cm in the WT to 111.31 ± 0.66 cm in *oslsd3-1* and 107.05 ± 0.81 cm in *oslsd3-2*, corresponding to increases of 8.72% and 4.56%, respectively (Figure 5a). In contrast, the panicle length of *oslsd3-1* decreased by 7.26%, from 23.04 ± 0.38 cm in the WT to 21.37 ± 0.21 cm (Figure 5b).

The 1000-grain weight increased by 9.58% in *oslsd2-1* and by 8.74% in *oslsd3-1* compared with the WT (Figure 5c). Grain length was also significantly increased in *oslsd3-1* and *oslsd4-1*, reaching 7.68 ± 0.05 and 7.70 ± 0.04 mm, respectively, compared with 7.43 ± 0.06 mm in the WT (Figure 5d). The longer-grain phenotypes of *oslsd3-1* and *oslsd4-1* were also visually apparent in the mature-grain comparison (Figure 5e). Collectively, increased plant height was consistently observed in both oslsd3 mutant lines, supporting a reproducible OsLSD3-associated effect on plant height. In contrast, the changes in 1000-grain weight, grain length, and panicle length were detected in individual mutant lines and should therefore be considered line-specific observations pending confirmation with additional independent alleles or complementation.

### 2.10. Subcellular Localization of OsLSD2, OsLSD3, and OsLSD4

**Figure 6:**
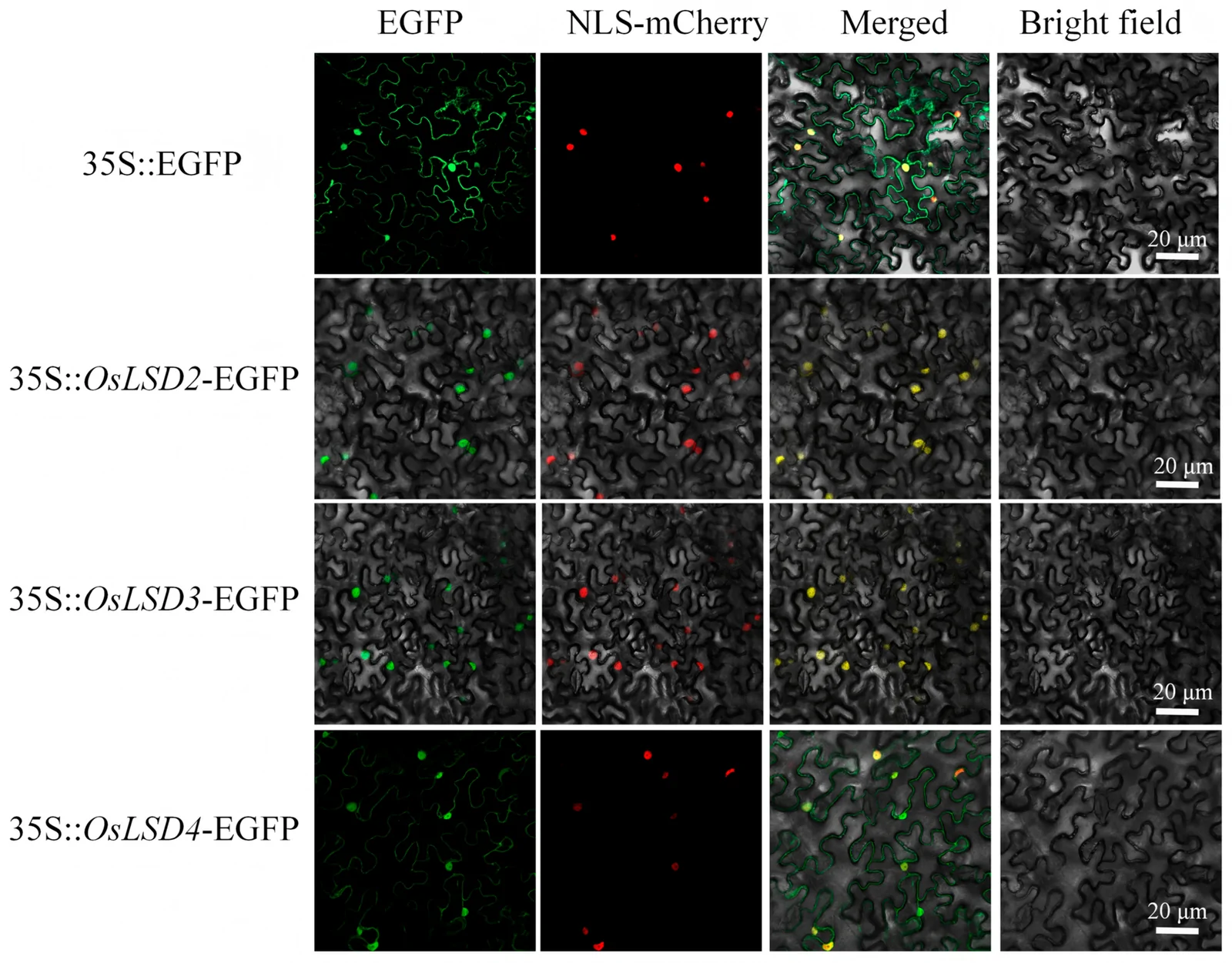
Subcellular localization of OsLSD2, OsLSD3, and OsLSD4 in Nicotiana benthamiana leaf epidermal cells. The coding sequences of OsLSD2, OsLSD3, and OsLSD4 without stop codons were fused in-frame with EGFP and transiently expressed in *N. benthamiana* leaf epidermal cells under the control of the CaMV 35S promoter. The empty 35S::EGFP vector was used as the control, and NLS-mCherry was co-expressed as a nuclear marker. Free EGFP fluorescence was distributed throughout the cytoplasm and nucleus. The fluorescence signals of OsLSD2-EGFP and OsLSD3-EGFP largely overlapped with the NLS-mCherry signal, indicating predominant nuclear localization. OsLSD4-EGFP was predominantly localized in the nucleus, with additional weak fluorescence detected in the cytoplasm. From left to right, the columns show EGFP fluorescence, NLS-mCherry fluorescence, merged images, and bright-field images. Images were captured 72 h after agroinfiltration. Scale bars = 20 μm.

To determine the intracellular distribution patterns of rice LSD1-like family members, OsLSD2, OsLSD3, and OsLSD4 were selected for subcellular localization analysis. The fluorescence signal of the empty PBI121-EGFP vector was diffusely distributed in both the nucleus and cytoplasm. Fluorescence imaging showed that the green fluorescence signals of OsLSD2-EGFP and OsLSD3-EGFP largely overlapped with the nuclear marker and were mainly concentrated in the nucleus, indicating that OsLSD2 and OsLSD3 are nuclear-localized proteins. The fluorescence signal of OsLSD4-EGFP was predominantly detected in the nucleus, with additional weak signals observed in the cytoplasm, suggesting that OsLSD4 exhibits predominant nuclear localization with partial cytoplasmic distribution (Figure 6).

## 3. Discussion

### 3.1. Evolutionary Conservation and Diversification of the Rice LSD1-like Family

The present study provides an integrated view of the rice LSD1-like gene family by combining comparative genomics, promoter and expression analyses, natural variation analysis, subcellular localization, and loss-of-function phenotyping. Seven members were identified, consistent with the number reported in an earlier evolutionary survey of the rice LSD1-like family [18]. The principal advance of the present study is the integration of updated family-wide characterization with population-level natural variation and comparative loss-of-function phenotyping. The results revealed substantial divergence among rice LSD1-like genes in protein architecture, gene organization, regulatory features, expression behavior, and phenotypic contribution. Notably, OsLSD3 was supported by convergent evidence from haplotype analysis and two independent mutant lines, thereby prioritizing this gene for further mechanistic investigation. These findings are consistent with the hypothesis that evolutionary and regulatory divergence within the rice LSD1-like family is accompanied by functional specialization.

The multi-species phylogeny separated rice LSD1-like proteins into two principal lineages. The broad taxonomic representation of Group 1, including bryophyte, basal-angiosperm, monocot, and dicot homologs, is consistent with an ancient and relatively conserved lineage. By contrast, the restriction of Group 2 to grasses suggests either lineage-specific expansion and divergence in Poaceae or differential loss in other plant lineages. The presence of several rice-maize collinear pairs, together with the absence of detectable rice-Arabidopsis collinearity, further supports stronger conservation within grasses. Nevertheless, the absence of a collinear pair should not be interpreted as evidence that a homolog or function is absent, because extensive genome rearrangement and sequence divergence can obscure ancient synteny. Within rice, only the OsLSD2-OsLOL1 pair was retained as a detectable collinear pair, indicating that large-scale duplication alone does not explain the current family composition.

Structural variation among family members may be functionally important. OsLSD2, OsLOL1, and OsLSD3 shared a compact and similar motif arrangement, whereas OsLOL5 contained a much longer protein sequence and several additional motifs. Because Arabidopsis LSD1 is thought to act as a condition-dependent scaffold and transcriptional co-regulator rather than as a conventional sequence-specific transcription factor [11], regions outside the core LSD1-like zinc-finger domains may influence partner selection, protein stability, or regulatory context. Interactions of LSD1 with bZIP10 and catalases demonstrate that distinct protein interfaces can connect LSD1 activity with transcriptional control and redox homeostasis [13,14]. The marked differences in exon-intron structure and accessory motifs observed here therefore provide plausible structural bases for functional specialization, although the contributions of individual motifs will require domain-deletion and protein-interaction experiments.

### 3.2. Regulatory and Expression Divergence Among Family Members

Predicted promoter cis-element profiles and transcript patterns further suggest regulatory diversification among family members. Light-responsive elements were broadly represented, whereas hormone- and stress-responsive elements varied substantially among genes. This pattern is consistent with the established role of LSD1-related proteins at the interface of light, reactive oxygen species, and defense signaling [10,11]. The distinct tissue and stress responses also agree with previous gene-specific studies in rice: OsLOL1 participates in gibberellin-dependent seed germination [20], OsLOL2 affects growth and disease resistance [21], and OsLOL5 contributes to saline–alkaline and oxidative-stress tolerance [22]. In the present dataset, OsLSD3 was preferentially expressed in roots and was repressed by cold, while OsLSD1, OsLOL2, and OsLSD4 were induced by heat but repressed by salt. These differences suggest that individual family members may respond to partially distinct upstream signals or function in different tissues and developmental stages.

These promoter and transcriptome analyses are hypothesis-generating: predicted cis-elements do not establish functional regulatory sites, and expression changes cannot distinguish direct from indirect stress responses. Nevertheless, together with the structural divergence, these patterns suggest functional diversification among rice LSD1-like members and provide testable hypotheses for future validation.

### 3.3. Natural Variation in OsLSD3 and Its Association with Plant Height

The OsLSD3 haplotype analysis revealed six haplotypes with pronounced differences in population composition. Hap I and Hap II were enriched in Japonica, whereas Hap III-Hap V were predominantly represented by Indica accessions. Strong haplotype stratification is expected in rice because major varietal groups have extensive genome-wide differentiation and distinct linkage-disequilibrium patterns [24]. Accordingly, the significant plant-height difference between Hap I and Hap II cannot by itself establish a causal effect of the seven haplotype-defining SNPs. Residual subpopulation structure, geographic adaptation, and linked loci may contribute to the observed difference even within the Japonica panel.

The position and linkage pattern of the lead GWAS signal are also informative. The plant-height-associated SNP was located approximately 140 kb upstream of OsLSD3 and showed only moderate linkage disequilibrium with three of the seven core SNPs. This configuration is compatible with several alternatives: the lead variant may regulate OsLSD3 at a distance, one of the linked haplotype SNPs may affect OsLSD3 expression or processing, or the association may be driven by another gene or regulatory element in the local interval [25]. Importantly, the taller Hap II accessions and the increased height of both oslsd3 mutants are directionally consistent with a possible role of OsLSD3 in the negative regulation of plant height. This convergence increases the biological plausibility of the association but still falls short of identifying a causal natural allele. Local association analysis with population covariates, expression quantitative trait locus mapping, promoter-activity assays, and precise allele replacement will be required to resolve causality.

### 3.4. Mutant Phenotypes and Potential Agronomic Roles

The two independent *oslsd3* mutant lines both showed increased plant height, providing the most reproducible phenotypic evidence in this study. In *oslsd3-1,* the height increase was accompanied by greater 1000-grain weight and grain length but reduced panicle length. Such multi-trait effects are not unexpected for regulators of rice architecture, because changes in developmental signaling can improve one yield component while compromising another; OsSPL14/IPA1 is a well-established example of an architecture regulator with pleiotropic effects on tillering, panicle traits, lodging resistance, and yield [26]. However, because the grain- and panicle-related changes observed in *oslsd3-1* were not detected in *oslsd3-2*, increased plant height represents the most reproducible OsLSD3-associated phenotype in the present study. The changes in 1000-grain weight, grain length, and panicle length should therefore be regarded as line-specific until independently validated.

The increases in 1000-grain weight in *oslsd2-1* and *oslsd3-1* and the longer grains of *oslsd3-1* and *oslsd4-1* suggest that multiple LSD1-like members may influence grain development. Classical rice grain-size loci such as GW2, GS3, and GL7 regulate grain width, length, and weight through distinct molecular mechanisms [26,27]. The present data do not place OsLSD2, OsLSD3, or OsLSD4 within any of these established pathways, but they identify testable entry points for examining whether redox status, cell proliferation, cell elongation, or hormone signaling is altered in developing spikelets. Histological analysis of lemma and palea cells, stage-specific expression profiling, and genetic interaction tests with known grain-size genes would help determine which developmental process is affected.

From a breeding perspective, increased grain weight or length should not be interpreted directly as increased yield, particularly because both oslsd3 mutants were taller and *oslsd3-1* had shorter panicles. Because the current field evaluation was conducted in a single genetic background and in one season and location, with unequal plot replication among genotypes, the agronomic effects should be interpreted cautiously. Multi-environment trials with balanced plot-level replication, together with complementation or additional independent alleles, will be required to evaluate the agronomic value of these mutations.

### 3.5. Subcellular Localization, Mechanistic Implications, and Future Directions

Experimental localization showed that OsLSD2 and OsLSD3 were mainly nuclear, whereas OsLSD4 was predominantly nuclear with a weak cytoplasmic signal. These observations are compatible with a role in transcription-associated protein complexes and agree conceptually with the scaffold/co-regulator model established for Arabidopsis LSD1 [11,13]. The partial cytoplasmic distribution of OsLSD4 may indicate nucleocytoplasmic shuttling or compartment-dependent interactions. The discrepancy between experimental localization and several in silico predictions also illustrates the importance of validating localization empirically, particularly for small or structurally divergent proteins. However, because these localization assays relied on transient overexpression in Nicotiana benthamiana under the CaMV 35S promoter, they may not fully reproduce the native expression level, tissue context, or subcellular distribution of these proteins in rice. Localization analyses under native regulatory contexts or directly in rice tissues will therefore be useful for further validation.

A mechanistic link between the observed agronomic phenotypes and programmed cell death or redox regulation remains unresolved. Although rice LSD1-like proteins have been associated with metacaspase interaction networks [23] and Arabidopsis LSD1 is involved in ROS- and defense-related regulation [12,13,14,15,16,17] ROS status, cell-death responses, hormone levels, and downstream transcriptional changes were not examined in the present mutants. Future studies should prioritize complementation, targeted ROS and cell-death assays, and identification of OsLSD3 nuclear partners to determine whether its effects on plant height and grain traits involve redox or hormone signaling. Overall, the integrated evidence identifies OsLSD3 as the strongest candidate in this family for plant architecture and grain-related traits, with OsLSD2 and OsLSD4 representing additional candidates for grain-weight and grain-length regulation.

## 4. Materials and Methods

### 4.1. Identification of Rice LSD1-like Gene Family Members

The whole-genome protein sequences of rice (*Oryza sativa*, IRGSP-1.0) were downloaded from Ensembl Plants, and only the longest transcript isoform was retained for each alternatively spliced gene [28]. The hidden Markov model (HMM) profile of the conserved LSD1 family domain (PF06943) was obtained from the InterPro database [29]. Homology searches were performed on a Linux server using the hmmsearch program in HMMER, with an E-value threshold of 1 × 10^−5^ [30]. The candidate protein sequences were then submitted to NCBI Batch CD-Search for conserved domain validation [31]. Only proteins containing the conserved LSD1 domain were retained as final family members, and their official gene symbols recommended by the database were used for nomenclature. The physicochemical properties of LSD1-like proteins, including molecular weight (MW), theoretical isoelectric point (pI), instability index, and grand average of hydropathicity (GRAVY), were predicted using the ExPASy ProtParam (accessed 15 August 2025) tool [32]. Subcellular localization was predicted using the Plant-mPLoc (version 2.0) and WoLF PSORT (version 2.0) online tools, and the results from both platforms were integrated for analysis [33,34]. Signal peptides were predicted using SignalP 6.0 [35].

### 4.2. Construction of a Phylogenetic Tree of LSD1-like Family Members from Multiple Plant Species

Seven representative plant species were selected for analysis, including rice (*Oryza sativa*), Arabidopsis thaliana, maize (*Zea mays*), soybean (*Glycine max*), *Brachypodium distachyon*, *Amborella trichopoda*, and *Physcomitrium patens*. Whole-genome protein sequences of these species were downloaded from Ensembl Plants, and LSD1-like family members were identified using the same method described for rice. The identified LSD1-like protein sequences from the seven species were combined and aligned using MAFFT (version 7.526) [36]. A phylogenetic tree was constructed using FastTree (version 2.1.11) and subsequently visualized and annotated using the iTOL online platform [37,38].

### 4.3. Gene Structure and Conserved Motif Analysis

To analyze the gene structures and conserved motif compositions of rice LSD1-like family members, the GFF3 annotation file and protein sequences of the seven LSD1-like genes were extracted. Gene structure information was obtained from the GFF3 file and visualized using TBtools (version 2.096) [39]. Conserved motifs were identified using the MEME Web Server by submitting the protein sequences to the MEME online platform [40]. The phylogenetic tree, MEME results, and gene structure files were then imported into TBtools to generate an integrated diagram showing phylogenetic relationships, gene structures, and conserved motif distributions.

### 4.4. Chromosomal Localization and Collinearity Analysis

Based on the GFF3 annotation file of the rice reference genome IRGSP-1.0, the chromosomal locations and physical coordinates of the identified LSD1-like genes were extracted. The “Gene Location Visualize from GTF/GFF” and “One Step MCScanX” functions in TBtools were used to visualize the chromosomal distribution of LSD1-like genes and to perform intraspecific collinearity analysis [39,41].

For interspecific collinearity analysis, rice was used as the reference species, while Arabidopsis and maize were selected as comparison species. The “One Step MCScanX-Super Fast” function in TBtools was used to identify homologous collinear blocks based on protein sequences and genome annotation files from each species. Collinear gene pairs associated with LSD1-like family members were then screened and visualized using TBtools [39,41].

### 4.5. Prediction and Visualization of Promoter Cis-Acting Elements

Based on the rice reference genome IRGSP-1.0 and its GFF3 annotation file, the 2000 bp upstream sequences from the start codon (ATG) of each LSD1-like gene were extracted using TBtools and used as promoter regions. These sequences were submitted to the PlantCARE database for cis-acting element prediction [42]. The predicted elements were filtered by removing basic transcription-related elements, and elements with clear functional annotations were retained. These elements were classified into four categories: phytohormone-responsive, abiotic stress-responsive, plant growth and development-related, and light-responsive elements. Finally, the distribution of cis-acting elements was visualized using the Basic Biosequence View function in TBtools, and the number of elements in each category was calculated.

### 4.6. Expression Pattern Analysis of Rice LSD1-like Genes

Expression profiles of rice LSD1-like genes were obtained from the Rice Genome Annotation Project (RGAP) database. Transcript abundance, expressed as transcripts per million (TPM), was collected from different tissues, and abiotic stress treatments were assessed, including seedling heat stress and multiple developmental tissues (Table S7). Expression values were visualized using the Heatmap module in TBtools. The TPM values were log2-transformed before visualization, and the heatmap was used to display relative expression patterns of OsLSD/OsLOL genes [43].

### 4.7. Natural Variation and Haplotype Analysis of OsLSD3

Natural variation data for OsLSD3 (LOC_Os08g03610/Os08g0130100) were obtained from RiceVarMap v3.0 using the Os-Nipponbare-Reference-IRGSP-1.0 genome assembly [44]. The queried interval was chr08:1,685,254–1,690,804, covering the OsLSD3 gene body and approximately 1 kb downstream and 2 kb upstream regions. Because OsLSD3 is located on the reverse strand, higher genomic coordinates correspond to the transcriptional upstream region.

A total of 110 variants were identified. Simple biallelic SNPs with deletion frequencies below 5% and estimated minor allele frequencies of at least 0.10 were retained. Variants in untranslated, splice, intronic, and upstream regions were prioritized. Seven representative core SNPs were selected: vg0801686408, vg0801686892, vg0801687667, vg0801688081, vg0801689418, vg0801689852, and vg0801690354.

Haplotype analysis was performed for 4666 rice accessions using the RiceVarMap Haplotype Network Analysis module. Accessions were classified as Indica, Japonica, Aus, or Intermediate. Associations between the four major haplotypes and population groups were evaluated using Pearson’s chi-square test, with Cramér’s V used to estimate effect size. The low-frequency Hap V and Hap VI were included only in descriptive analyses.

Plant-height GWAS signals were retrieved from the RiceVarMap Japonica panel. Linkage disequilibrium between the lead SNP vg0801829820 and the seven core SNPs was assessed in 1497 Japonica accessions. Plant height was compared between 109 Hap I and 95 Hap II Japonica accessions using Welch’s two-sample *t*-test, with the Mann–Whitney U test used as a robustness analysis. All tests were two-sided [45].

### 4.8. Mutant Acquisition, Genotyping, and Agronomic Trait Measurement

CRISPR/Cas9-edited materials of OsLOL1, OsLSD2, OsLSD3, and OsLSD4 were purchased from Biogle Biotechnology Co., Ltd. (Hangzhou, China; https://www.seedseek.cn). All edited materials were generated in the rice cultivar Zhonghua 11 (ZH11) background. The mutant lines were self-pollinated to the T2 generation, and homozygous mutants were identified by genotyping. Homozygous plants without residual Cas9/T-DNA vector sequences were selected for subsequent phenotypic analysis. For genotyping, genomic DNA was extracted from leaf tissues using the CTAB method. The edited target regions were amplified using gene-specific primers and subjected to Sanger sequencing. The sequencing results were aligned with the wild-type sequences using SnapGene (version 7.2.1) to determine the mutation types and homozygous status. Only the intended CRISPR/Cas9 target regions were genotyped by Sanger sequencing; potential off-target sites were not systematically assessed in these mutant lines.

The T_2_ materials were planted in an experimental field in Wuxi, Jiangsu Province, in May 2025. Wild-type ZH11 and mutant lines were grown in plots. Because the numbers of available homozygous plants differed among mutant lines, each genotype was planted in 1–3 independent plots, with 36 plants per plot. The plant spacing and row spacing were 20 cm and 30 cm, respectively. Field management followed local standard rice cultivation practices. The detailed sample sizes for each genotype and trait are provided in Table S9.

Agronomic traits were investigated at maturity. To reduce border effects, plants at the edges of each plot were excluded, and phenotypically uniform plants were randomly selected from the central area of each plot. The measured traits included plant height, productive tiller number, panicle length, grain length, grain width, 1000-grain weight, and seed setting rate. Plant height was measured from the plant base to the tip of the highest panicle. Productive tiller number was defined as the number of tillers bearing mature panicles per plant. Panicle length was measured from the panicle base to the panicle tip. Grain length and grain width were measured using mature grains. The 1000-grain weight was determined using fully mature and filled grains. Seed setting rate was calculated as the percentage of filled grains relative to the total number of grains.

All data are presented as mean ± standard error (SE). Statistical analyses were performed using SPSS Statistics 26.0 (IBM Corp., Armonk, NY, USA). Because the numbers of plots and effective sampled plants differed among genotypes, one-way analysis of variance (ANOVA) under an unbalanced design was used to evaluate differences in agronomic traits among genotypes. Dunnett’s test was then performed using wild-type ZH11 as the control. Statistical significance was set at *p* < 0.05. Figures were generated using OriginPro 2023 (OriginLab Corporation, Northampton, MA, USA).

### 4.9. Subcellular Localization Analysis of OsLSD2, OsLSD3, and OsLSD4

To determine the subcellular localization of OsLSD2, OsLSD3, and OsLSD4, total RNA was extracted from leaves of ZH11 rice plants and reverse-transcribed into complementary DNA (cDNA). The full-length coding sequences of OsLSD2, OsLSD3, and OsLSD4 without stop codons were amplified from cDNA to ensure in-frame fusion with the EGFP tag. The amplified fragments were cloned into the XbaI-linearized pBI121-EGFP vector by homologous recombination, generating C-terminal EGFP fusion constructs driven by the CaMV 35S promoter. Positive recombinant plasmids were verified by Nanopore sequencing using primers designed from the CaMV 35S promoter region to confirm the insertion direction, sequence integrity, and correct reading frame. The verified constructs were named 35S::OsLSD2-EGFP, 35S::OsLSD3-EGFP, and 35S::OsLSD4-EGFP.

The recombinant plasmids were transformed into Agrobacterium tumefaciens strain GV3101 and transiently expressed in leaves of 4-week-old Nicotiana benthamiana plants. The nuclear marker construct 35S::NLS-mCherry was co-transformed to label nuclei, and the empty pBI121-EGFP vector was used as a negative control. Each fusion construct was tested in at least three independent biological replicates. At 72 h after infiltration, fluorescence signals in the infiltrated leaf regions were observed using a laser scanning confocal microscope. EGFP fluorescence, NLS-mCherry fluorescence, and bright-field images were collected. The subcellular localization of OsLSD2, OsLSD3, and OsLSD4 was determined based on the overlap between EGFP signals and the nuclear marker.

## 5. Conclusions

This study identified seven LSD1-like genes in rice and demonstrated that the family combines a conserved zinc-finger core with substantial evolutionary, structural, and regulatory divergence. Phylogenetic and collinearity analyses resolved conserved and grass-associated lineages, while promoter and expression profiles indicated tissue- and stress-dependent specialization. Natural variation analysis of OsLSD3 revealed six strongly population-structured haplotypes, and two common Japonica haplotypes differed in plant height. Consistently, two independent oslsd3 mutants were taller than the wild type, supporting increased plant height as the most reproducible OsLSD3-associated phenotype. Other agronomic changes were line-specific: *oslsd3-1* showed increased 1000-grain weight and grain length but reduced panicle length, whereas the single *oslsd2-1* and *oslsd4-1* lines showed increased 1000-grain weight and grain length, respectively. Because mutant phenotyping was conducted in one genetic background and in a single season and location, these agronomic effects require validation in additional environments and independent alleles. OsLSD2 and OsLSD3 localized mainly to the nucleus, and OsLSD4 showed predominant nuclear localization with a weak cytoplasmic signal. Together, these findings prioritize OsLSD3 for functional dissection of plant architecture and grain development and identify OsLSD2 and OsLSD4 as additional agronomic candidates. Causal natural variants, molecular partners, and the underlying redox or hormone mechanisms remain to be validated before these genes can be considered for rice improvement.

## Figures and Tables

**Table 1:** Frequency and population distribution of OsLSD3 haplotypes among 4666 rice accessions.

Haplotype	Sequence	*n*	Frequency	Indica	Aus	Japonica	Intermediate	Dominant Population	Dominant %
Hap I	TCACAGA	943	20.21%	24	1	836	82	Japonica	88.65%
Hap II	TTTCCGA	879	18.84%	31	200	613	35	Japonica	69.74%
Hap III	CCAGCGG	1352	28.98%	1266	66	7	13	Indica	93.64%
Hap IV	CCAGCAG	1351	28.95%	1294	1	30	26	Indica	95.78%
Hap V	CCTGCGG	113	2.42%	107	0	0	6	Indica	94.69%
Hap VI	TTTCAGA	28	0.60%	10	0	14	4	Japonica	50.00%

Note: Haplotypes were identified and accession assignments were obtained using the Haplotype Network Analysis module of RiceVarMap v3.0 based on the allelic combinations of seven representative core SNPs ordered from SNP1 to SNP7. The “Sequence” column shows the corresponding seven-SNP allelic combination. n indicates the number of accessions assigned to each haplotype, and frequency was calculated relative to the total of 4666 accessions. The Indica, Aus, Japonica, and Intermediate columns show the numbers of accessions from the population categories predefined in RiceVarMap v3.0. “Dominant population” indicates the most abundant population within each haplotype, and “Dominant %” represents its proportion among all accessions assigned to that haplotype.

**Table 2:** Comparison of plant height between Japonica accessions carrying OsLSD3 Hap I and Hap II.

Group	*n*	Mean	SD	Median
Hap I	109	123.63	33.83	115.80
Hap II	95	149.68	19.39	150.20

Note: Japonica accessions were classified into Hap I and Hap II using the haplotype assignments generated by the Haplotype Network Analysis module of RiceVarMap v3.0. n indicates the number of accessions included in each haplotype group. Mean, standard deviation (SD), and median plant height are expressed in centimeters. Differences between Hap I and Hap II were evaluated using Welch’s two-sample *t*-test, with the Mann–Whitney U test used as a nonparametric robustness analysis. All tests were two-sided.

**Table 3:** Sequence alignment of CRISPR/Cas9 target regions and mutation types in rice LSD1-like mutant lines.

Target Gene	sgRNA	Allele	Aligned Sequence (5′–3′)	Mutation Type	Indel Size (bp)
OsLOL1	OsLOL1 -sgRNA1	WT	GGGTGCAGGAACATTCTGCTCTACCCGAGA**GGG**GCACCG	Reference	0
		*oslol1-1*	GGGTGCAGGAACATTCTGCTCTACCC--AGAGGGGCACCG	Deletion	−1
		*oslol1-2*	GGGTGCAGGAACATTCTGCTCTACCCG**A**AGAGGGGCACCG	Insertion	1
	OsLOL1 -sgRNA2	WT	CTGCGATACAGTCAATCTTGTCAGACC**AGG**TACTTGCAT	Reference	0
		*oslol1-3*	CTGCGATACAGTCAATCTTGT---------------------------------AT	Deletion	−16
OsLSD2	OsLSD2 -sgRNA	WT	CCGGAGCGTGCTGCGGTACCCGAG**CGG**GGCGCCC	Reference	0
		*oslsd2-1*	CCGGAGCGTGCTGCGGTACCCGA**A**GCGGGGCGCCC	Insertion	1
OsLSD3	OsLSD3 -sgRNA	WT	GGGCAGTGC**CGG**ACGACTCTGATGCACCCACCTGGA	Reference	0
		*oslsd3-1*	GGGCAGTGCCGGAC**G**GACTCTGATGCACCCACCTGGA	Insertion	1
		*oslsd3-2*	GGGCAGTGCCGGAC**C**GACTCTGATGCACCCACCTGGA	Insertion	1
OsLSD4	OsLSD4 -sgRNA	WT	TCATCACACTGTCCTAGAGC**CGG**GTCGCCCCAGTGA	Reference	0
		*oslsd4-1*	TCATCACACTGTCCTAG----------------------------------TGA	Deletion	−16

Note: WT indicates the wild-type Zhonghua 11 (ZH11) reference sequence. Aligned sequences are shown in the 5′–3′ orientation. Underlining indicates sgRNA target sequences, bold letters indicate PAM sequences, and bold underlined letters indicate inserted nucleotides relative to the WT sequence. Hyphens indicate deleted nucleotides. Mutation types and indel sizes were determined by Sanger sequencing of homozygous mutant lines. Negative and positive indel values indicate deletions and insertions, respectively. OsLOL1 was targeted by two sgRNAs, whereas OsLSD2, OsLSD3, and OsLSD4 were each targeted by one sgRNA.

**Table 4:** Significant differences in agronomic traits between wild-type Zhonghua 11 and rice LSD1-like mutant lines.

Trait	Mutant Line	WT, *n*	WT Mean ± SE	Mutant, *n*	Mutant Mean ± SE	Difference (Mutant − WT)	Change (%)	95% CI of Difference	Dunnett-Adjusted *p* Value	Significance
Plant height (cm)	*oslsd3-1*	6	102.38 ± 1.20	12	111.31 ± 0.66	8.925	8.717	5.11 to 12.74	1.58 × 10^−7^	***
Plant height (cm)	*oslsd3-2*	6	102.38 ± 1.20	12	107.05 ± 0.81	4.667	4.558	0.86 to 8.48	1.04 × 10^−2^	*
1000-grain weight (g)	*oslsd2-1*	6	23.14 ± 0.52	5	25.36 ± 0.39	2.217	9.579	0.55 to 3.88	4.50 × 10^−3^	**
1000-grain weight (g)	*oslsd3-1*	6	23.14 ± 0.52	12	25.17 ± 0.26	2.023	8.742	0.65 to 3.40	1.38 × 10^−3^	**
Grain length (mm)	*oslsd3-1*	6	7.43 ± 0.06	12	7.68 ± 0.05	0.247	3.320	0.05 to 0.44	8.42 × 10^−3^	**
Grain length (mm)	*oslsd4-1*	6	7.43 ± 0.06	12	7.70 ± 0.04	0.268	3.600	0.07 to 0.46	3.70 × 10^−3^	**
Panicle length (cm)	*oslsd3-1*	6	23.04 ± 0.38	12	21.37 ± 0.21	−1.673	−7.263	−3.00 to −0.35	7.63 × 10^−3^	**

Note: WT indicates the wild-type rice cultivar Zhonghua 11 (ZH11). Values are presented as mean ± SE, and n indicates the sample size used in the statistical analysis. Only mutant–WT comparisons showing significant differences after multiple-comparison adjustment are presented. Differences, percentage changes, and 95% confidence intervals were calculated relative to WT, with differences expressed as mutant minus WT. Positive and negative values indicate increases and decreases, respectively. Statistical significance was determined by one-way ANOVA followed by a two-sided Dunnett’s test using WT as the control. * *p* < 0.05, ** *p* < 0.01, and *** *p* < 0.001.

## Data Availability

The data supporting the findings of this study are available within the article and its Supplementary Materials. The raw agronomic trait data and statistical analysis results are provided in Tables S9 and S10, respectively. The RNA-seq TPM expression data analyzed in this study are provided in Table S7, and the sources of publicly available datasets are described in Section 4.

## Supplementary Materials

The following supporting information can be downloaded at: https://www.mdpi.com/article/10.3390/plants15172554/s1, Table S1: Physicochemical properties and predicted subcellular localization of LSD1-like proteins identified in rice; Table S2: Summary of HMMER screening and conserved domain validation for rice LSD1-like family members; Table S3: LSD1-like proteins from multiple species used for phylogenetic analysis; Table S4: Conserved motifs identified in rice LSD1-like proteins; Table S5: Chromosomal locations and intra- and inter-species collinearity relationships of rice LSD1-like genes; Table S6: Cis-acting elements in the promoters of rice LSD1-like genes; Table S7: RNA-seq TPM expression profiles of rice LSD1-like genes; Table S8: Primers used for mutant validation; Table S9: Raw agronomic trait data of ZH11 and mutant lines; Table S10: Statistical analysis of agronomic traits in ZH11 and mutant lines; Table S11: Primers used for subcellular localization vector construction; Table S12: Information and selection rationale for the seven selected SNPs in the gene body and upstream region; Table S13: Haplotype assignments, haplotype sequences, and plant height of 204 accessions.
